# Independent and synergistic associations of sleep quality and frailty with cardiometabolic multimorbidity: a cross-sectional analysis in Chinese elderly population

**DOI:** 10.1186/s12877-026-07985-8

**Published:** 2026-07-16

**Authors:** Siyu Bing, Weida Liu, Shuting Song, Yujiao Dong, Shuwen Mao, Qian Tang, Aihong Wang

**Affiliations:** 1 School of Nursing, Shandong Second Medical University, Weifang, 261053 China; 2 Peking Union Medical College Hospital, State Key Laboratory for Complex Severe and Rare Diseases, Institute of Clinical Medicine, Chinese Academy of Medical Sciences, Beijing, 100000 China; 3 Affiliated Hospital of Shandong Second Medical University, Weifang, 261033 China; 4 Shandong University of Traditional Chinese Medicine Affiliated Hospital, Jinan, 250000 China

**Keywords:** Aged, Sleep quality, Frailty, Cardiovascular Diseases, Metabolic Diseases, Multimorbidity

## Abstract

**Background:**

Cardiometabolic multimorbidity (CMM) poses a substantial public health burden in aging populations. However, limited evidence exists regarding the clustering of cardiometabolic conditions or the interplay between sleep and frailty in CMM. This study aimed to explore the independent and synergistic associations of these factors with CMM, which may inform targeted preventive strategies.

**Methods:**

This cross-sectional study analyzed data from the 2018 wave of the Chinese Longitudinal Healthy Longevity Survey (CLHLS). Self-reported sleep quality was categorized as good, fair, or bad. Frailty was quantified using a 35-item deficit accumulation frailty index (FI), dichotomized as non-frail (FI ≤ 0.25) or frail (FI > 0.25). Multivariable logistic regression models assessed associations between sleep quality, frailty, and CMM (≥ 2 concurrent cardiometabolic conditions). Multiplicative interaction was tested via likelihood ratio tests, while additive interaction was quantified using the relative excess risk due to interaction (RERI), attributable proportion (AP), and synergy index (SI).

**Results:**

Among 8,592 participants (mean age 83.17 ± 11.00 years; 48.0% male), CMM prevalence was 21.6% (*n* = 1,854). Females exhibited higher CMM prevalence than males (54.7% vs. 45.3%), with significant sex- and age-specific disparities (e.g., 65–74 years: females 28.1% vs. males 21.3%; 75–84 years: females 28.5% vs. males 23.5%; all *P* < 0.05). In adjusted models, bad sleep quality was independently associated with higher odds of CMM (bad vs. good: adjusted odds ratio [aOR] = 1.84, 95% CI: 1.55–2.18; fair vs. good: aOR = 1.22, 95% CI: 1.06–1.40), with a dose-response relationship (*P* < 0.001). Frailty demonstrated the strongest association (frail vs. non-frail: aOR = 6.87, 95% CI: 5.70–8.28). Notably, frail individuals with bad sleep quality had 9.18-fold higher odds of CMM (95% CI: 6.63–12.72) compared to non-frail good sleepers, with 21% of CMM cases attributable to this interaction (RERI = 1.09, 95% CI: 0.08–2.10; AP = 0.21, 95% CI: 0.06–0.36; SI = 1.35, 95% CI: 1.03–1.67).

**Conclusions:**

Sleep quality and frailty are associated with CMM, suggesting that their combined assessment may enhance predictive accuracy for CMM risk. These findings underscore the need to prioritize high-risk elderly individuals for integrated interventions addressing sleep disturbances and frailty, thereby facilitating the transition from reactive to proactive cardiometabolic care models.

**Supplementary Information:**

The online version contains supplementary material available at https://doi.org/10.1186/s12877-026-07985-8.

## Introduction

Cardiometabolic multimorbidity (CMM)—defined as the coexistence of two or more cardiometabolic disorders (e.g., cardiovascular disease (CVD), dyslipidemia, type 2 diabetes)—represents a growing public health challenge, particularly among aging populations [1, 2]. In China, the prevalence of multimorbidity in individuals aged ≥ 60 years ranges from 6.4% to 76.5%, with cardiometabolic conditions accounting for a disproportionate burden of disease severity [3]. Age-related physiological decline, including vascular stiffening, endothelial dysfunction, and metabolic dysregulation, is associated with increased susceptibility to clustered cardiometabolic pathologies in older adults [4]. The interplay between these conditions is associated with exacerbated cardiovascular risk, is associated with accelerated functional decline, and imposes substantial socioeconomic costs through direct medical expenditures and indirect disability-related burdens.

Sleep, a vital physiological process in humans, is closely associated with cardiometabolic disorders [5]. Emerging evidence links bad sleep quality—characterized by shortened duration, fragmented patterns, or circadian misalignment—to dysregulated metabolic homeostasis and heightened cardiovascular risk [6]. Mechanistically, chronic sleep loss may be associated with altered hormone secretion and immune system function, which work together to create a proinflammatory and prothrombotic environment [7]. Notably, obstructive sleep apnea (OSA), a common sleep-related breathing disorder, exemplifies this pathophysiological link, with intermittent hypoxia during apnea episodes may trigger endothelial damage and metabolic disturbances, which are thought to increase the risk of adverse cardiac events [8].

Frailty, a geriatric syndrome marked by diminished physiological reserve across multiple domains, further compounds cardiometabolic vulnerability in older adults. Frail older adults have decreased physical reserve capacity and diminished resistance to various stressors, all of which act in synergy with cardiometabolic disorders to perpetuate the vicious cycle of physical decline [9]. Previous studies have shown that frail older adults often suffer from multiple chronic diseases, of which cardiometabolic diseases are an important component [10]. However, the existing literature mainly focused on isolated cardiometabolic diseases rather than clusters of comorbidities, which has constrained the development of comprehensive management strategies for CMM in older adults [11].

However, the interplay between sleep quality and frailty in shaping CMM remains underexplored. While both factors are independently associated with adverse outcomes, their potential synergistic effects—mediated through shared pathways of oxidative stress, inflammation, and autonomic dysfunction—merit population-based investigation [12–14]. This study addresses this knowledge gap by examining the independent and joint associations of sleep quality and frailty with CMM in a nationally representative sample of Chinese older adults. By elucidating these relationships, we aim to inform targeted interventions that mitigate the compounding risks of sleep dysfunction, frailty, and cardiometabolic decay in aging populations.

## Methods

### Data source

This cross-sectional study utilized the data from the 2018 wave of the Chinese Longitudinal Healthy Longevity Survey (CLHLS), a nationally representative study that was initiated in 1998 and focuses on healthy aging determinants. As the largest publicly available geriatric dataset in China, the CLHLS employs a multi-stage stratified cluster sampling design across 23 provinces (representing ~ 85% of the national population) with deliberate oversampling of individuals aged ≥ 90 years to enhance statistical power for underrepresented oldest-old subgroups. The data collection integrates standardized face-to-face interviews, clinical assessments, and biospecimen collection, encompassing comprehensive indicators such as sociodemographics (age, sex, education), socioeconomic status (income, marital status), health-related behaviors (smoking, physical activity), and physiological biomarkers (blood pressure, heart rate) [15].

### Study population

**Fig. 1:**
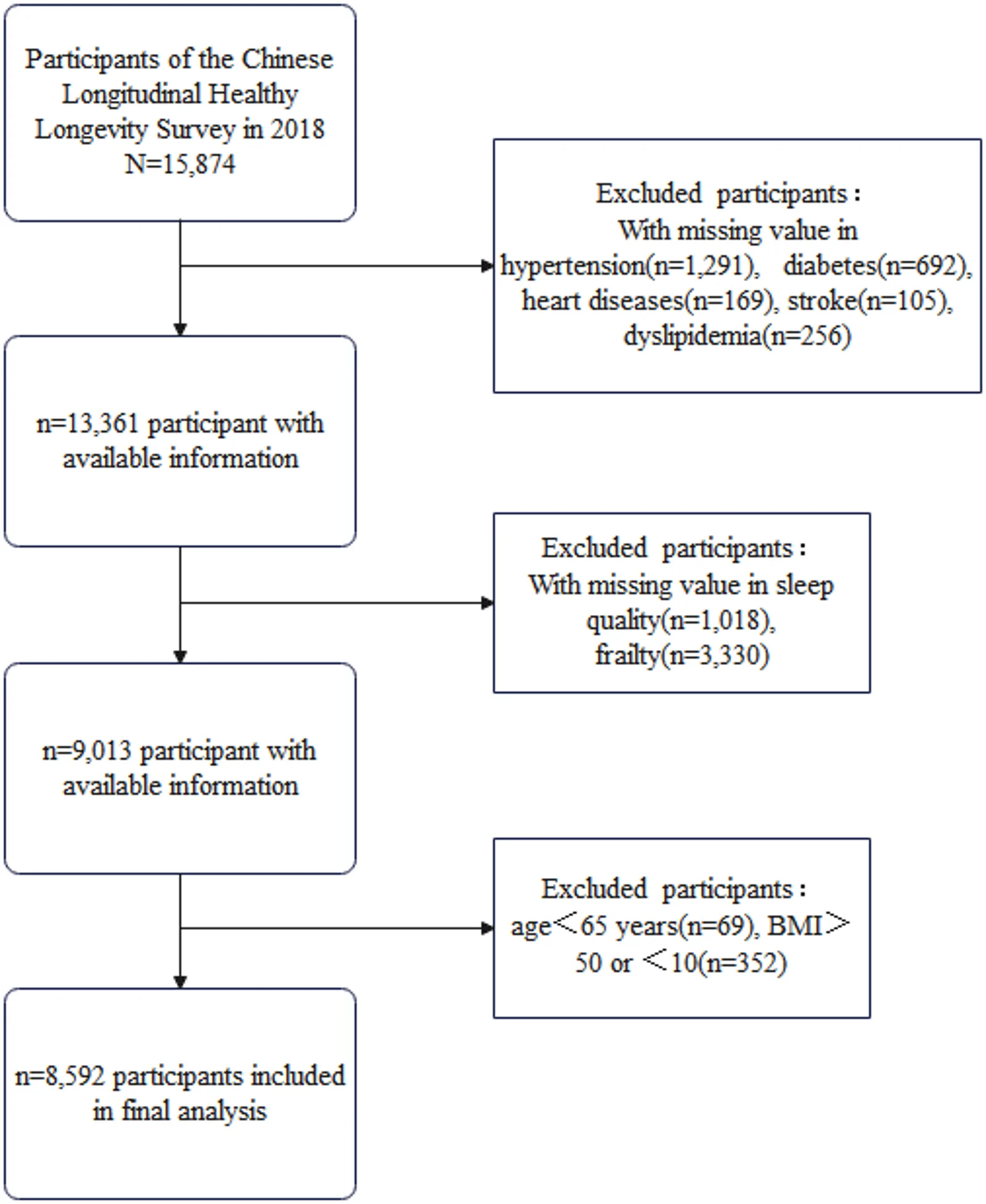
Flowchart for selecting the research population

Of 15,874 eligible participants in the 2018 CLHLS, we applied exclusion criteria sequentially: (1) missing data on key exposures (sleep quality, frailty) or outcome (CMM); (2) age < 65 years (to align with WHO elderly classification); and (3) biologically implausible covariate values (e.g., BMI < 10 kg/m²or > 50 kg/m²). The final analysis sample included 8,592 participants. Figure 1 shows the flowchart for selecting the study population.

### Exposure assessments

#### Sleep quality

Sleep quality was self-reported via the single-item question: “How would you rate your current sleep quality?” with responses categorized as: (1) good (very good/good), (2) fair (so-so), or (3) bad (bad/very bad). This approach balances participant burden with predictive validity, as demonstrated by strong correlations with multi-item sleep scales in prior validation studies [16, 17].

#### Frailty

Frailty was quantified using a 35-item Frailty Index (FI) constructed from CLHLS health deficits [18, 19]. These variables include health condition, self-care ability, daily activities and habits, psychology and attitude, senses and bodily functions, and disease condition [20]. Most of the items have binary options, with a score of 0 (no) or 1 (yes). Some items, such as self-rated health status, are classified as healthy, moderately healthy, fair, unhealthy, and very unhealthy, with scores of 0, 0.25, 0.5, 0.75, and 1, respectively. The FI was calculated as the proportion of accumulated deficits relative to total items, with participants classified as frail (FI > 0.25) versus non-frail (FI ≤ 0.25) per established thresholds [21].

### Outcome assessment

#### Cardiometabolic multimorbidity, CMM

CMM was defined as the co-occurrence of ≥ 2 conditions from: hypertension, diabetes mellitus, heart disease, stroke, or dyslipidemia [1]. Although some studies define CMM more narrowly as the coexistence of heart disease, stroke, and diabetes, we adopted a broader definition including hypertension and dyslipidemia, which is consistent with several large-scale epidemiological studies [22, 23]. This approach reflects the full spectrum of cardiometabolic burden in very old adults; however, we acknowledge that definitional heterogeneity may limit direct comparability with studies using a narrower definition. Disease diagnoses were based on standardized self-reported physician confirmation, with all five cardiometabolic conditions assessed via the same question format: ‘Has a doctor ever told you that you are suffering from [condition]?’ Response options were ‘yes,’ ‘no,’ or ‘don’t know’—participants who answered ‘yes’ were classified as having the condition, ‘no’ as not having it, and ‘don’t know’ as missing data. Individual cardiometabolic conditions were analyzed as secondary outcomes.

#### Covariates

Demographic characteristics and health-related variables were included as covariates, including age (65 to 74, 75 to 84, 85 to 94, or 95 and older), sex (male or female), educational attainment (elementary school and below, secondary school, and college and above), occupation (farmer or non-farmer), place of residence (city, town, or rural), marital status (married and cohabiting or others), body mass index (BMI) (according to the Chinese-specific cutoffs recommended by the Working Group on Obesity in China: underweight < 18.5, normal 18.5–23.9, overweight 24–27.9, obesity ≥ 28 kg/m²), living arrangement (living with house member (s), living alone, or in an institution), smoking status (yes or no), alcohol consumption status (yes or no), and exercise status (yes or no).

### Statistical analysis

All analyses were performed using SAS 9.4. In descriptive statistics, categorical variables were expressed as frequencies (%), and continuous variables were reported as mean ± standard deviation (SD). Group comparisons were analyzed using chi-square tests to assess differences in the proportion of CMM across sleep quality categories (good/fair/bad) and frailty status (yes/no). Fisher’s exact test was applied when the expected cell count was < 5.

To address confounding effects, three-level nested binary logistic regression models were constructed: Model 1 (unadjusted) estimated the crude odds ratios (ORs) for the associations of sleep quality and frailty with CMM; Model 2 (demographically adjusted) controlled for age and sex (male/female), which were considered common confounders in older populations; Model 3 (fully adjusted) further adjusted for socioeconomic factors (education level: primary/secondary/tertiary education; occupation: farmer/non-farmer) and lifestyle variables (BMI, smoking status, etc.).

Multiplicative interactions were assessed via likelihood ratio tests (LRTs) of product terms (sleep quality × frailty). To comprehensively evaluate additive interaction between sleep quality and frailty, three complementary epidemiological indicators were adopted in the present study. The Relative Excess Risk due to Interaction (RERI) quantifies the excess disease risk attributable to interaction beyond the independent effects of each factor, with a RERI > 0 indicating a positive additive interaction. The Attributable Proportion (AP) reflects the proportion of disease risk among individuals exposed to both risk factors that can be attributed to interaction, where an AP > 0 suggests that a considerable proportion of cases could be avoided if the interactive effect was eliminated. The Synergy Index (SI) calculates the ratio of the observed joint effect to the expected additive effect, and an SI > 1 denotes the presence of synergistic effects. Collectively, reporting these three indicators enables a comprehensive and robust characterization of additive interaction, as each metric has distinct interpretations and varying sensitivities to effect size and baseline disease risk.

Additive interaction was further quantified using the above three indicators. Bootstrap resampling (1,000 iterations) generated 95% CIs for interaction metrics, with the exclusion of zero indicating statistical significance. Variables with missing values underwent multiple imputations (5 datasets) with Rubin’s rules for pooling. Sensitivity analyses confirmed the robustness of the primary findings. Hosmer-Lemeshow tests (*P* > 0.10) confirmed adequate model fit for all regression analyses. Two-sided *P* < 0.05 was taken to indicate statistical significance.

## Results

### Baseline characteristics

Table 1 summarizes baseline characteristics stratified by CMM status. Significant between-group differences were observed across demographic, clinical, and behavioral domains (all *P* < 0.05). Participants in the CMM group were predominantly aged 65–84 years (65–74 years: 29.2% vs. 24.7%; 75–84 years: 37.1% vs. 29.1%), whereas the non-CMM group had a higher proportion of individuals aged ≥ 95 years (20.8% vs. 9.2%). Compared to the non-CMM group, the CMM group exhibited higher rates of being married, female, urban residents, elevated body mass index (BMI), and physical inactivity (all *P* < 0.001).

**Table 1:** Characteristics of the study population

Variable	Total(*N* = 8592)	CMM group (*n* = 1854)	Non-CMM group (*n* = 6738)	*P*-value
Age, No. (%)				<0.001
65–74	2206(25.6)	541(29.2)	1665(24.7)	
75–84	2651(30.9)	688(37.1)	1963(29.2)	
85–94	2161(25.2)	455(24.5)	1706(25.3)	
≥ 95	1574(18.3)	170(9.2)	1404(20.8)	
Sex, No. (%)				0.007
male	4126(48.0)	839(45.3)	3287(48.8)	
female	4466(52.0)	1015(54.7)	3451(51.2)	
Marital status, No. (%)^a^				<0.001
married	3902(45.8)	952(51.6)	1950(44.2)	
partnered/single/widowed	4617(54.2)	893(48.4)	3724(55.8)	
Residence, No. (%)				<0.001
city	1975(23.0)	784(42.3)	1191(17.7)	
town	2858(33.3)	488(26.3)	2370(35.2)	
rural	3759(43.7)	582(31.4)	3177(47.1)	
Body Mass Index (BMI), No. (%)^a^				<0.001
<18.5	1226(14.5)	111(6.1)	1115(16.8)	
18.5–24	4436(52.5)	809(44.5)	3627(54.6)	
24–28	2079(24.6)	651(35.8)	1428(21.5)	
>28	712(8.4)	245(13.6)	467(7.1)	
Co-residence, No. (%)^a^				<0.001
living with house member(s)	6860(80.8)	1478(80.5)	5382(80.8)	
living alone	1409(16.6)	286(15.6)	1123(16.9)	
in an institution	224(2.6)	72(3.9)	152(2.3)	
Smoking, No. (%)^a^				<0.001
yes	1412(16.6)	217(11.8)	1195(17.9)	
no	7101(83.4)	1616(88.2)	5485(82.1)	
Drinking, No. (%)^a^				<0.001
yes	1359(16.0)	189(10.3)	1170(17.6)	
no	7138(84.0)	1647(89.7)	5491(82.4)	
Physical activity, No. (%)^a^				<0.001
yes	3011(35.5)	796(43.3)	2215(33.3)	
no	5481(64.5)	1042(56.7)	4439(66.7)	
Occupation, No. (%)^a^				<0.001
non-farmer	2854(39.3)	966(57.9)	1888(33.7)	
farmer	4409(60.7)	701(42.1)	3708(66.3)	
Education, No. (%)^a^				<0.001
primary	5682(78.0)	1089(65.5)	4593(81.7)	
secondary	1308(18.0)	425(25.5)	883(15.7)	
tertiary	294(4.0)	149(9.0)	145(2.6)	

*CMM* Cardiometabolic multimorbidity

^a^ Missing data: 73 participants for marital status (0.8%), 139 for body mass index (1.6%), 99 for co-residence (1.2%), 79 for smoking (0.9%), 95 for drinking (1.1%), 100 for physical activity (1.2%), 1329 for occupation (15.5%), 1308 for education (15.2%). The multiple imputation dataset is shown in Supplementary Table 4

### Age- and sex-specific disease prevalence

**Fig. 2:**
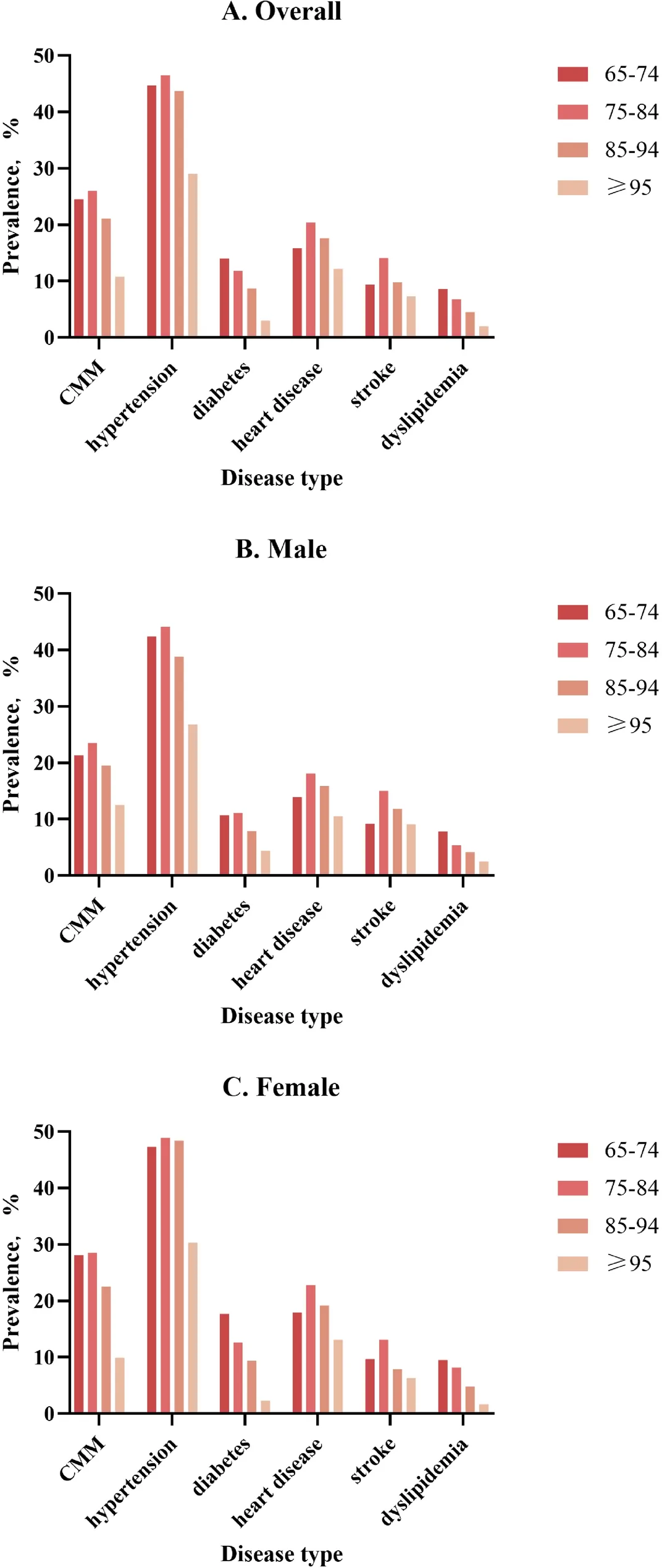
Gender-specific cardiometabolic multimorbidity and the prevalence of its components at different age groups. Note: (**A**) Overall population; (**B**) Male participants; (**C**) Female participants. Bars represent the prevalence (%) of each cardiometabolic disorder stratified by four age groups: 65–74 years, 75–84 years, 85–94 years, and ≥95 years

As shown in Fig. 2 and Table S1, age- and sex-stratified disease prevalence analyses revealed notable variations. In the overall population, the prevalence of CMM peaked in the 75–84 age group (26.0%), while hypertension prevalence also reached its highest level in this group (46.5%). In contrast, diabetes, cardiovascular disease (CVD), stroke, and dyslipidemia prevalence displayed declining trends with advancing age. Sex-specific differences were evident: females consistently exhibited higher CMM prevalence across all age groups compared to males, particularly in the 65–74 age group (28.1% vs. 21.3%, *P*<0.001), though males showed marginally higher CMM prevalence in the ≥ 95 age group (12.5% vs. 9.9%, *P* = 0.111).

### Sleep quality and cardiometabolic multimorbidity risk

Table 2 details the independent associations between sleep quality and CMM. As shown in Table 2, bad sleep quality was associated with significantly higher odds of CMM compared with good sleep quality, with a clear dose-response trend (P for trend < 0.001). All component diseases of CMM showed positive correlations with sleep quality, with all P for trend < 0.001.

**Table 2:** The association between sleep quality and cardiometabolic multimorbidity

Outcome	Events/No.	Model 1		Model 2		Model 3	
		OR(95%CI)	*P*-value	OR(95%CI)	*P*-value	OR(95%CI)	*P*-value
Cardiometabolic multimorbidity							
Sleep quality			<0.001		<0.001		<0.001
good	893/4662	Reference		Reference		Reference	
fair	579/2691	1.16(1.03–1.30)	0.02	1.13(1.00−1.27)	0.05	1.22(1.06–1.40)	0.01
bad	382/1239	1.88(1.63–2.17)	<0.001	1.80(1.56–2.08)	<0.001	1.84(1.55–2.18)	<0.001
Continuous	1854/8592	1.33(1.24–1.43)	<0.001	1.30(1.21–1.40)	<0.001	1.33(1.23–1.44)	<0.001
Hypertension							
Sleep quality			<0.001		<0.001		<0.001
good	1867/4662	Reference		Reference		Reference	
fair	1138/2691	1.10(1.00−1.21)	0.06	1.06(0.96–1.17)	0.22	1.16(1.04–1.30)	0.01
bad	616/1239	1.48(1.31–1.68)	<0.001	1.41(1.24–1.61)	<0.001	1.43(1.23–1.66)	<0.001
Continuous	3621/8592	1.19(1.12–1.26)	<0.001	1.16(1.09–1.23)	<0.001	1.19(1.11–1.27)	<0.001
Diabetes							
Sleep quality			<0.001		<0.001		<0.001
good	415/4662	Reference		Reference		Reference	
fair	281/2691	1.19(1.02–1.40)	0.03	1.16(0.99–1.36)	0.08	1.32(1.10–1.58)	<0.001
bad	160/1239	1.52(1.25–1.84)	<0.001	1.44(1.1781.75)	<0.001	1.37(1.09–1.72)	0.01
Continuous	856/8592	1.23(1.12–1.35)	<0.001	1.19(1.08–1.31)	<0.001	1.20(1.07–1.33)	<0.001
Heart disease							
Sleep quality			<0.001		<0.001		<0.001
good	694/4662	Reference		Reference		Reference	
fair	462/2691	1.19(1.04–1.35)	0.01	1.15(1.01–1.30)	0.04	1.26(1.09–1.47)	<0.001
bad	306/1239	1.88(1.61–2.18)	<0.001	1.78(1.52–2.07)	<0.001	1.83(1.54–2.19)	<0.001
Continuous	1462/8592	1.34(1.24–1.44)	<0.001	1.30(1.21–1.40)	<0.001	1.34(1.23–1.46)	<0.001
Stroke							
Sleep quality			<0.001		<0.001		<0.001
good	450/4662	Reference		Reference		Reference	
fair	286/2691	1.11(0.95–1.30)	0.18	1.13(0.97–1.32)	0.13	1.08(0.90–1.29)	0.40
bad	171/1239	1.50(1.24–1.81)	<0.001	1.52(1.25–1.84)	<0.001	1.58(1.28–1.95)	<0.001
Continuous	907/8592	1.20(1.10–1.32)	<0.001	1.21(1.11–1.33)	<0.001	1.23(1.10–1.36)	<0.001
Dyslipidemia							
Sleep quality			<0.001		<0.001		<0.001
good	249/4662	Reference		Reference		Reference	
fair	144/2691	1.00(0.81–1.24)	0.99	0.97(0.79–1.21)	0.81	1.04(0.81–1.32)	0.78
bad	105/1239	1.64(1.29–2.08)	<0.001	1.55(1.22–1.98)	<0.001	1.73(1.32–2.26)	<0.001
Continuous	498/8592	1.23(1.10–1.39)	0.001	1.20(1.06–1.36)	<0.001	1.27(1.11–1.45)	<0.001

Model 1 no adjusted, Model 2 adjusted for age and sex, Model 3 further adjusted for place of residence, educational attainment, living arrangement, marital status, occupation, smoking, drinking, exercise, and body mass index. The ‘Continuous’ row tests for linear trend across sleep quality categories (Good, Fair, Bad) when modeled as an ordinal continuous variable; the corresponding p-value is the P for trend

### Frailty and cardiometabolic multimorbidity risk

Frailty demonstrated the strongest independent association with CMM (Table 3). Frailty was strongly associated with CMM in all models. In the fully adjusted model (Model 3), frail individuals had significantly higher odds of CMM compared with non‑frail individuals (see Table 3 for detailed OR and 95% CI).

**Table 3:** The association between frailty and cardiometabolic multimorbidity

Outcome	Events/No.	Model 1		Model 2		Model 3	
		OR(95%CI)	*P*-value	OR(95%CI)	*P*-value	OR(95%CI)	*P*-value
Cardiometabolic multimorbidity							
Frailty			<0.001		<0.001		<0.001
non-frail	1329/7399	Reference		Reference		Reference	
frail	525/1193	3.59(3.16–4.08)	<0.001	6.39(5.48–7.45)	<0.001	6.87(5.70–8.28)	<0.001
Hypertension							
Frailty			<0.001		<0.001		<0.001
non-frail	2942/7399	Reference		Reference		Reference	
frail	679/1193	2.00(1.77–2.27)	<0.001	2.73(2.39–3.13)	<0.001	2.74(2.34–3.21)	<0.001
Diabetes							
Frailty			<0.001		<0.001		<0.001
non-frail	642/7399	Reference		Reference		Reference	
frail	214/1193	2.30(1.94–2.72)	<0.001	3.97(3.29–4.79)	<0.001	3.82(3.07–4.77)	<0.001
Heart disease							
Frailty			<0.001		<0.001		<0.001
non-frail	1042/7399	Reference		Reference		Reference	
frail	420/1193	3.32(2.89–3.80)	<0.001	4.26(3.76–5.07)	<0.001	4.18(3.50–4.99)	<0.001
Stroke							
Frailty			<0.001		<0.001		<0.001
non-frail	575/7399	Reference		Reference		Reference	
frail	332/1193	4.58(3.93–5.33)	<0.001	6.75(5.68–8.01)	<0.001	6.82(5.58–8.35)	<0.001
Dyslipidemia							
Frailty			<0.001		<0.001		<0.001
non-frail	400/7399	Reference		Reference		Reference	
frail	98/1193	1.57(1.24–1.97)	<0.001	2.48(1.94–3.17)	<0.001	2.28(1.70–3.05)	<0.001

Model 1 no adjusted, Model 2 adjusted for age and sex, Model 3 further adjusted for place of residence, educational attainment, living arrangement, marital status, occupation, smoking, drinking, exercise, and body mass index

### Synergistic effects of sleep quality and frailty

**Fig. 3:**
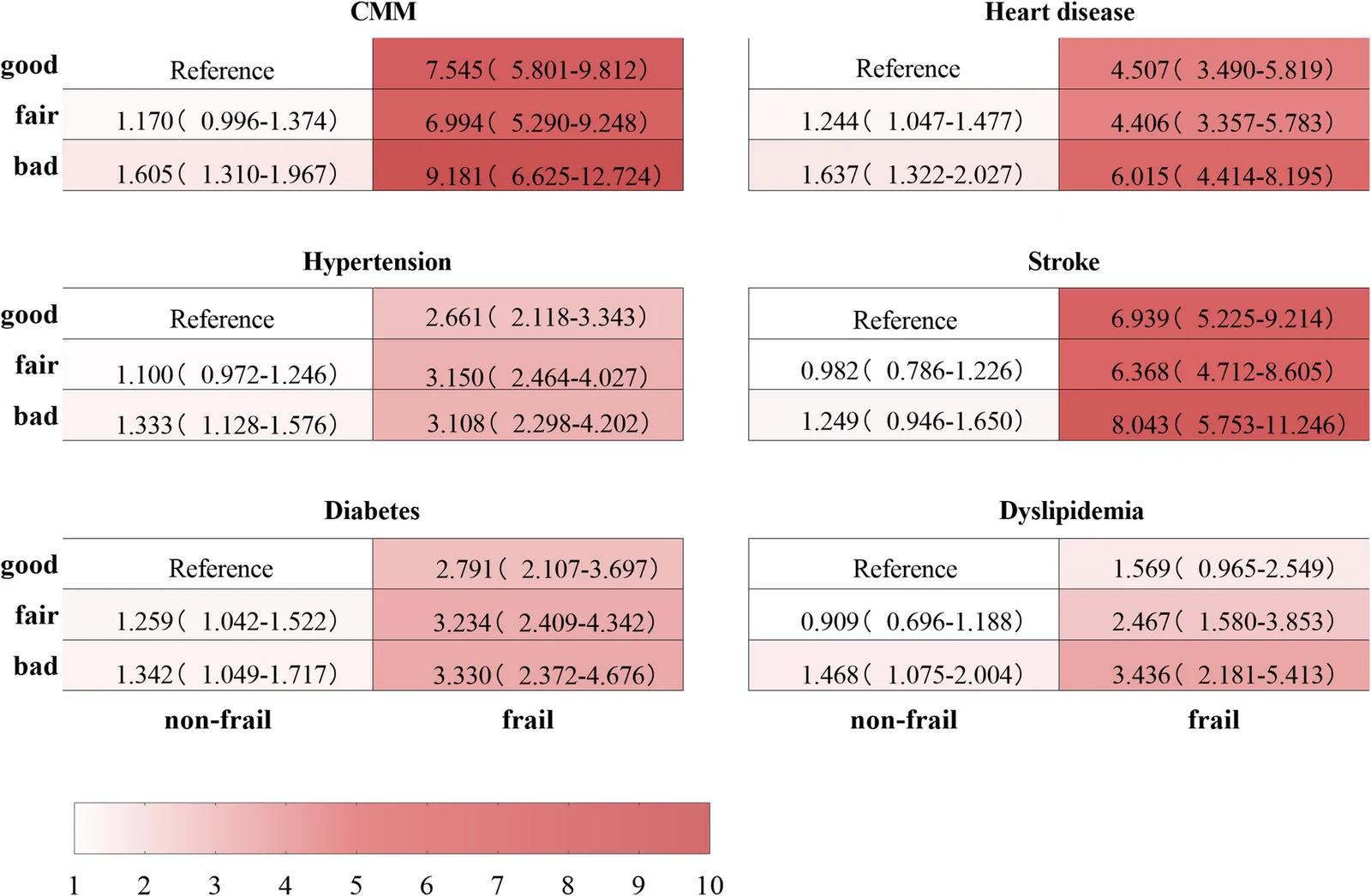
The combined effect of sleep quality and frailty on cardiometabolic multimorbidity and its components Note: The dataset of multiple imputation is shown in Supplementary Table 5

Figure 3 illustrates the combined impact of sleep quality and frailty on CMM risk. The joint effect of bad sleep quality and frailty on CMM was substantially greater than the sum of their individual effects. As illustrated in Fig. 3, frail participants with bad sleep quality had the highest odds of CMM, and additive interaction analysis confirmed significant synergy (see Table 4). Additive interaction analysis (Table 4) revealed significant synergy for CMM (RERI = 1.09, 95% CI: 0.08–2.10; SI = 1.35, 95% CI: 1.03–1.67), with 21% (AP = 0.21, 95% CI: 0.06–0.36) of CMM cases attributable to sleep-frailty interactions. Notably, similar significant additive interactions were simultaneously observed in heart disease, with RERI = 1.00 (95% CI: 0.00–2.00), AP = 0.21 ( 95% CI: 0.04–0.37), and SI = 1.35 (95% CI: 1.00–1.70), further supporting the consistency of this synergistic pattern across specific cardiovascular outcomes. In contrast, no statistically significant additive interactions were detected for other CMM components (e.g., hypertension, diabetes), suggesting that the interplay between sleep quality and frailty may be selectively relevant to certain cardiovascular and metabolic phenotypes.

**Table 4:** The effects of sleep quality and frailty on the addition and multiplication of cardiometabolic multimorbidity and its components

	*P* for interaction	RERI(95%CI)	AP(95%CI)	SI(95%CI)
CMM	<0.001	1.09(0.08,2.10)	0.21(0.06,0.36)	1.35(1.03,1.67)
Heart disease	<0.001	1.00(0.00,2.00)	0.21(0.04,0.37)	1.35(1.00,1.70)
Stroke	<0.001	0.45(−1.06,1.96)	0.09(−0.18,0.35)	1.12(0.72,1.52)
Hypertension	<0.001	0.13(-0.25,0.51)	0.05(-0.09,0.20)	1.10(0.82,1.38)
Diabetes	<0.001	0.23(-0.48,0.95)	0.08(-0.15,0.31)	1.15(0.70,1.59)
Dyslipidemia	<0.001	0.51(−0.18,1.20)	0.21(−0.01,0.42)	1.53(0.86,2.19)

*CMM* Cardiometabolic multimorbidity, *RERI* Relative Excess Risk due to Interaction, *AP* Attributable Proportion, *SI* Synergy Index

Sensitivity analyses were performed to verify the robustness of the main findings, which were consistent in the full data set (with 8,592 participants) and the multiple-imputation data set (Tables S2 and S3). In stratified analyses (Table S6), the association between frailty and CMM remained strong and statistically significant across both sexes and age groups. No significant interactions were detected for sex (*P* = 0.68) or age group (*P* = 0.45), indicating that the effect of frailty on CMM does not differ significantly by these demographic factors.

## Discussion

This population-based study elucidates the independent and synergistic associations of sleep quality and frailty with CMM in Chinese older adults. Our findings suggest that both sleep dysfunction and frailty are strongly associated with CMM, and their combined presence correlates with amplified cardiometabolic risk. Given the cross-sectional design, these associations should be interpreted as correlational rather than causal.

### Sleep quality as a cardiometabolic modulator

Good sleep quality was associated with lower CMM prevalence., consistent with previous evidence linking sleep health to metabolic-cardiovascular homeostasis. Sebastian, S.A.‘s research found that individuals with high sleep quality have a 27% lower risk of CHD and a 19% lower risk of stroke, likely attributed to the stabilization of sympathetic nervous activity during sleep, which maintains physiological parameters such as blood pressure and heart rate at optimal levels, thereby reducing cardiovascular strain [24]. Conversely, our study revealed a dose-response relationship between bad sleep quality and elevated CMM risk, with exhibiting 1.84-fold higher odds compared to good sleepers (P for trend < 0.001). This aligns with mechanistic insights showing that fragmented sleep patterns and circadian misalignment may impair glucose homeostasis through elevated interleukin-6 (IL-6) and C-reactive protein (CRP) levels, which may be associated with accelerated atherosclerosis and insulin resistance [25]. Chronic sleep deprivation is further associated with disrupted autonomic balance, favoring sympathetic overactivation and nocturnal blood pressure surges, which may be associated with exacerbated vascular stiffness—a hallmark of age-related cardiometabolic decline. These findings extend prior observations from single-disease studies, such as the 1.33-fold increased hypertension risk and 1.18-fold elevated heart disease risk associated with insufficient sleep, to the context of multimorbidity [26]. The bidirectional relationship underscores that while restorative sleep is associated with lower cardiometabolic burden through anti-inflammatory and autonomic stabilization pathways, chronic sleep disruption may contribute to a pro-thrombotic and pro-inflammatory milieu, which could increase CMM susceptibility in aging populations.

### Association of frailty with accelerated cardiometabolic aging

The strong association between frailty and CMM suggests that frailty is associated with systemic physiological stress, characterized by impaired musculoskeletal integrity, chronic inflammation (e.g., IL-6, TNF-α), and endocrine dysregulation that collectively foster cardiometabolic dysfunction [27]. In our study, frailty demonstrated the strongest independent association with CMM (adjusted OR = 6.87), consistent with cohort studies reporting associations between frailty indices and accelerated biological aging and mitochondrial dysfunction [28, 29], as well as meta-analyses reporting a 2.0-fold higher cardiovascular mortality risk in frail individuals [11].

Frailty-associated sarcopenia is associated with reduced metabolic flexibility, while chronic inflammation is associated with impaired endothelial repair and insulin resistance [30]. These mechanisms may be further associated with bidirectional interactions between energy dysregulation and functional decline. For instance, our findings revealed that frail individuals exhibited higher rates of obesity (BMI ≥ 24 kg/m²: 49.4% vs. 28.6% in the non-CMM group) and physical inactivity (56.7% vs. 66.7%), suggesting that adipose tissue accumulation and sedentary behavior may be associated with amplified inflammatory cascades and frailty progression [31]. Shakya et al.’s combined analysis corroborates this interplay, demonstrating that hypertension, diabetes, and frailty risk are interconnected through pathways involving chronic inflammation and oxidative stress [32]. Notably, previous studies report a fourfold higher odds of frailty in diabetic populations, while cross-sectional data reveal a 58% greater frailty prevalence among those with ≥ 5 cardiometabolic comorbidities compared to non-multimorbid individuals [27, 33].

These findings suggest a cyclical relationship of frailty and CMM in aging populations, where impaired glucose regulation is associated with cardiometabolic deterioration and IL-6-mediated oxidative damage is associated with accelerated frailty progression [34]. The clinical urgency to address multimorbidity as a unified entity is underscored by strong interrelationships between chronic disease burden and frailty indices, particularly in diabetic patients. Our results indicate that frailty is closely linked to and may exacerbate cardiometabolic deterioration, highlighting the need for integrated interventions targeting inflammatory pathways and metabolic resilience.

Stratified analyses revealed that the association between frailty and CMM was significantly modified by both sex and age. The stronger association in males suggests that frail older men may be particularly vulnerable to developing cardiometabolic multimorbidity, possibly due to sex‑specific differences in body composition, inflammatory profiles, or hormonal regulation. More notably, the markedly higher odds ratio in the < 85 years group (aOR = 9.87) compared with the ≥ 85 years group (aOR = 4.52) indicates that frailty is a more potent risk marker for CMM in the younger elderly, while its relative impact diminishes in the oldest‑old. One plausible explanation is that in very old individuals, competing risks (e.g., cognitive decline, functional dependence, polypharmacy) increasingly dominate the disease pathway, attenuating the incremental contribution of frailty to CMM. Another possibility is survivor bias, as the healthiest individuals survive into the oldest‑old age, leading to an underestimation of the frailty–CMM association. These findings highlight the need for age‑ and sex‑specific prevention strategies based on the observed associations.

### Synergistic associations of sleep-frailty interactions

This study further revealed the joint effects of sleep quality and frailty on CMM, with frail individuals experiencing bad sleep quality demonstrating 9.18-fold higher odds of CMM compared to non-frail good sleepers, underscoring the clinical relevance of this synergistic relationship. The additive interaction analysis (RERI = 1.09, AP = 0.21) highlights shared pathophysiological pathways driving this synergy. Mechanistically, sleep disruption is associated with amplified oxidative stress via NADPH oxidase activation, which synergizes with frailty-related mitochondrial dysfunction to exacerbate vascular endothelial damage [35]. Concurrently, physiological stress responses associated with bad sleep quality—including sympathetic overactivation and inflammatory cytokine release (e.g., IL-6, TNF-α)—are associated with aggravated functional decline in frail individuals, which may create a bidirectional vicious cycle [14, 36].

Conversely, chronic inflammation associated with frailty is associated with disrupted HPA axis regulation, which may further fragment sleep architecture and perpetuate metabolic dysregulation [37]. This aligns with experimental models demonstrating that intermittent hypoxia (as seen in obstructive sleep apnea) and frailty-associated IL-6 elevation are associated with NF-κB pathway activation, which may accelerate atherosclerosis progression [38]. Notably, the interaction exhibited selectivity for cardiovascular outcomes (e.g., heart disease: SI = 1.35), while no significant additive interaction was observed for hypertension, diabetes, stroke, or dyslipidemia alone.

This distinct disease-specific pattern warrants careful interpretation. One possible explanation is that the synergistic effects of poor sleep quality and frailty are most pronounced on cardiovascular outcomes that involve autonomic dysregulation and endothelial dysfunction—key pathological mechanisms shared by both sleep disruption and frailty. Heart disease (particularly coronary artery disease) is highly sensitive to cumulative inflammatory burden (e.g., IL-6, CRP) and sympathetic overactivation, which are markedly exacerbated when sleep disturbance and frailty co-occur. In contrast, hypertension and diabetes are predominantly driven by independent risk factors such as excessive sodium intake and insulin resistance, which may require longer exposure durations for interactive effects to emerge. Stroke and dyslipidemia rely on distinct etiological pathways, including large-vessel atherosclerosis and lipid metabolic disorders, which are less susceptible to synergistic amplification induced by the coexistence of poor sleep and frailty. Additionally, the non-significant interactions for individual chronic conditions may be partially attributed to limited statistical power, as several single outcomes had relatively small case numbers (e.g., dyslipidemia, *n* = 498). Therefore, we caution against overinterpreting the lack of synergy for specific diseases and emphasize that the significant interaction detected for composite CMM represents our most robust and reliable finding.

In our research sample, 21% of the association between sleep-frailty interaction and CMM was attributable to the interaction between sleep and frailty, indicating that intervention measures targeting both aspects may bring significant clinical benefits. These findings are consistent with cross-sectional observational results on elevated frailty burden among multi-diseased older adults and further expand current evidence by quantifying the incremental risk derived from concurrent sleep dysfunction [27, 33]. By elucidating the mechanistic links among chronic inflammation, oxidative stress, sleep disruption and frailty, our study provides a comprehensive theoretical framework. It also suggests that integrated intervention strategies combining sleep optimization with resistance training and physical conditioning may effectively interrupt the cardiovascular and metabolic pathological cascade in vulnerable elderly populations.

### Strengths and limitations

This study relied on a nationally representative CLHLS survey with a large-scale urban and rural sample, which enhanced the generalizability of findings. By analyzing independent and joint effects of sleep quality and frailty on CMM, we revealed synergistic risks beyond single-exposure patterns. The inclusion of CMM as an outcome aligns with the clinical complexity of overlapping chronic conditions in elderly populations. However, reliance on self-reported measures may introduce misclassification bias. Notably, our definition of CMM included hypertension and dyslipidemia, whereas some previous studies restricted CMM to heart disease, stroke, and diabetes. This broader diagnostic scope may increase the estimated prevalence of CMM in the current study and should be fully considered when comparing our results with those adopting narrower criteria. In addition, cognitively impaired older adults might underestimate symptoms or overestimate health status, potentially affecting the accuracy of our results.

The assessment of sleep quality in this study relied on a single self-reported item, which, while pragmatic for large-scale surveys, has several inherent limitations. First, single-item measures are susceptible to recall bias and fail to capture multidimensional sleep characteristics, including sleep duration, latency, fragmentation, and hypnotic medication use. Second, cultural disparities in sleep perception and self-reporting may exist among Chinese older adults, and social desirability bias as well as distinct emotional expression patterns could further influence their responses. Third, misclassification of sleep quality is inevitable, since individuals with undiagnosed sleep disorders (e.g., obstructive sleep apnea) may subjectively rate their sleep as satisfactory despite underlying pathological changes. Despite these shortcomings, single-item sleep quality tools have been widely adopted in aging cohort research and exhibit reliable predictive validity for multiple health outcomes. For example, Li et al. demonstrated that single-item-assessed sleep quality trajectories were strongly linked to new-onset diabetes mellitus among older adults [39]. Wang et al. revealed that alterations in single-item-measured sleep quality served as an independent predictor of later depressive symptoms in older Chinese adults [40]. Guo et al. further confirmed that single-item sleep quality partially mediated the long-term association between depressive symptoms and cognitive function over a 10-year follow-up [41]. Collectively, these evidence support the rationality and applicability of single-item sleep evaluation in large-scale geriatric studies. Nevertheless, our sleep-related findings should be interpreted with caution, and future investigations are warranted to adopt comprehensive and standardized sleep scales (e.g., the Pittsburgh Sleep Quality Index) for more accurate evaluation. Furthermore, as a cross-sectional study, our design cannot rule out reverse causality, and temporality and causal relationships between variables cannot be firmly established. Accordingly, all observed associations should be recognized as statistical correlations rather than definitive causal effects. Finally, despite the national representativeness of the CLHLS cohort, our results may not be generalized to non-Chinese populations, younger individuals, or regions with different healthcare and social welfare systems.

## Conclusions

Both sleep quality and frailty are independently associated with CMM risk in older Chinese adults, and their combined assessment may provide better predictive value for risk stratification than single-domain evaluations. The significant additive interaction detected for CMM and heart disease indicates that older adults with coexisting poor sleep and frailty may represent a high-risk subgroup that could benefit most from integrated targeted interventions. However, due to the cross-sectional nature of this study, causal inferences cannot be drawn.

Future longitudinal investigations are needed to clarify the temporal sequence and causal pathways underlying these associations, as well as to explore the condition-specific mechanisms of sleep–frailty interaction. In addition, well-designed interventional trials are warranted to verify whether targeted improvements in sleep quality and frailty status can effectively reduce CMM prevalence, especially among high-risk elderly populations. Collectively, our findings advocate for a paradigm shift in geriatric cardiometabolic care, shifting from traditional disease-specific management to comprehensive holistic bio-psycho-social interventions for vulnerable older adults.

Note: Model 1 no adjusted, Model 2 adjusted for age and sex, Model 3 further adjusted for place of residence, educational attainment, living arrangement, marital status, occupation, smoking, drinking, exercise, and body mass index. The ‘Continuous’ row tests for linear trend across sleep quality categories (Good, Fair, Bad) when modeled as an ordinal continuous variable; the corresponding p-value is the P for trend.

## Supplementary Information

Supplementary Material 1.

## Data Availability

The data can be obtained at (https://opendata.pku.edu.cn/dataverse/CHADS). Researchers must apply for permission to use the data.

## Abbreviations

aOR: adjusted Odds Ratio
AP: Attributable Proportion
BMI: Body Mass Index
CLHLS: Chinese Longitudinal Healthy Longevity Survey
CMM: Cardiometabolic Multimorbidity
CVD: Cardiovascular Disease
FI: Frailty Index
RERI: Relative Excess Risk due to Interaction
SI: Synergy Index
