# Supplementary material for: Independent and synergistic associations of sleep quality and frailty with cardiometabolic multimorbidity: a cross-sectional analysis in Chinese elderly population
Source: BMC Geriatr. 2026 Jul 16;26:1282. doi: 10.1186/s12877-026-07985-8 (PMC13656460; doi:10.1186/s12877-026-07985-8)
Supplement: Supplementary file 1 [file 12877_2026_7985_MOESM1_ESM.docx]

**Supplementary file:**

**Independent and Synergistic Associations of Sleep Quality and Frailty with Cardiometabolic Multimorbidity: A Cross-Sectional Analysis in Chinese Older People**

**Table S1.** Gender-specific cardiometabolic multimorbidity and the prevalence of its components at different age groups

**Table S2.** The association between sleep quality and cardiometabolic multimorbidity

**Table S3.** The association between frailty and cardiometabolic multimorbidity

**Table S4.** The characteristics of the research subjects after multiple fillings

**Table S5.** The combined effect of sleep quality and frailty on cardiometabolic multimorbidity and its components after multiple imputation

**Table S6.** Stratified analysis of the association between frailty and cardiometabolic multimorbidity (CMM) by sex and age group

**Table S1.** Gender-specific cardiometabolic multimorbidity and the prevalence of its components at different age groups(N=8592)

|  | **Overall** | **Male** | **Female** | **P-value** |
| --- | --- | --- | --- | --- |
| Cardiometabolic multimorbidity | | | | |
| Age, No. (%) | | | | |
| 65-74 | 541(24.5) | 248(21.3) | 293(28.1) | ＜0.001 |
| 75-84 | 688(26.0) | 314(23.5) | 374(28.5) | ＜0.001 |
| 85-94 | 455(21.1) | 206(19.5) | 249(22.5) | 0.09 |
| ≥95 | 170(10.8) | 71(12.5) | 99(9.9) | 0.11 |
| Hypertension | | | | |
| Age, No. (%) | | | | |
| 65-74 | 987(44.7) | 494(42.4) | 493(47.3) | 0.02 |
| 75-84 | 1232(46.5) | 590(44.1) | 642(48.9) | 0.01 |
| 85-94 | 945(43.7) | 409(38.8) | 536(48.4) | ＜0.001 |
| ≥95 | 457(29.0) | 153(26.8) | 304(30.3) | 0.15 |
| Diabetes | | | | |
| Age, No. (%) | | | | |
| 65-74 | 308(14.0) | 124(10.7) | 184(17.7) | ＜0.001 |
| 75-84 | 313(11.8) | 148(11.1) | 165(12.6) | 0.23 |
| 85-94 | 187(8.7) | 83(7.9) | 104(9.4) | 0.21 |
| ≥95 | 48(3.0) | 25(4.4) | 23(2.3) | 0.02 |
| Heart disease | | | | |
| Age, No. (%) | | | | |
| 65-74 | 348(15.8) | 162(13.9) | 186(17.9) | 0.01 |
| 75-84 | 542(20.4) | 242(18.1) | 300(22.8) | ＜0.001 |
| 85-94 | 380(17.6) | 168(15.9) | 212(19.2) | 0.05 |
| ≥95 | 192(12.2) | 60(10.5) | 132(13.1) | 0.13 |
| Stroke | | | | |
| Age, No. (%) | | | | |
| 65-74 | 208(9.4) | 107(9.2) | 101(9.7) | 0.69 |
| 75-84 | 373(14.1) | 201(15.0) | 172(13.1) | 0.16 |
| 85-94 | 211(9.8) | 124(11.8) | 87(7.9) | ＜0.001 |
| ≥95 | 115(7.3) | 52(9.1) | 63(6.3) | 0.04 |
| Dyslipidemia | | | | |
| Age, No. (%) | | | | |
| 65-74 | 190(8.6) | 91(7.8) | 99(9.5) | 0.16 |
| 75-84 | 180(6.8) | 72(5.4) | 108(8.2) | ＜0.001 |
| 85-94 | 97(4.5) | 44(4.2) | 53(4.8) | 0.49 |
| ≥95 | 31(2.0) | 14(2.5) | 17(1.7) | 0.30 |

**Table S2.** The association between sleep quality and cardiometabolic multimorbidity(N=8592)

| **Outcome** | **Events/No.** | **Model 1** |  | **Model 2** |  | **Model 3** |  |
| --- | --- | --- | --- | --- | --- | --- | --- |
|  |  | **OR(95%CI)** | **P-value** | **OR(95%CI)** | **P-value** | **OR(95%CI)** | **P-value** |
| Cardiometabolic multimorbidity | | | | | | | |
| Sleep quality |  |  | ＜0.001 |  | ＜0.001 |  | ＜0.001 |
| good | 893/4662 | Reference |  | Reference |  | Reference |  |
| fair | 579/2691 | 1.16(1.03-1.30) | 0.02 | 1.13(1.00-1.27) | 0.05 | 1.19(1.05-1.35) | 0.01 |
| bad | 382/1239 | 1.88(1.63-2.17) | ＜0.001 | 1.80(1.56-2.08) | ＜0.001 | 1.89(1.62-2.21) | ＜0.001 |
| Continuous | 1854/8592 | 1.33(1.24-1.43) | ＜0.001 | 1.30(1.21-1.40) | ＜0.001 | 1.340(1.30-1.38) | ＜0.001 |
| Hypertension | | | | | | | |
| Sleep quality |  |  | ＜0.001 |  | ＜0.001 |  | ＜0.001 |
| good | 1867/4662 | Reference |  | Reference |  | Reference |  |
| fair | 1138/2691 | 1.10(1.00-1.21) | 0.06 | 1.06(0.96-1.17) | 0.22 | 1.11(1.00-1.23) | 0.04 |
| bad | 616/1239 | 1.48(1.31-1.68) | ＜0.001 | 1.41(1.24-1.61) | ＜0.001 | 1.45(1.27-1.66) | ＜0.001 |
| Continuous | 3621/8592 | 1.19(1.12-1.26) | ＜0.001 | 1.16(1.09-1.23) | ＜0.001 | 1.18(1.15-1.22) | ＜0.001 |
| Diabetes | | | | | | | |
| Sleep quality |  |  | ＜0.001 |  | ＜0.001 |  | ＜0.001 |
| good | 415/4662 | Reference |  | Reference |  | Reference |  |
| fair | 281/2691 | 1.19(1.02-1.40) | 0.03 | 1.16(0.99-1.36) | 0.08 | 1.20(1.02-1.43) | 0.03 |
| bad | 160/1239 | 1.52(1.25-1.84) | ＜0.001 | 1.44(1.18-1.75) | ＜0.001 | 1.43(1.16-1.75) | ＜0.001 |
| Continuous | 856/8592 | 1.23(1.12-1.35) | ＜0.001 | 1.19(1.08-1.31) | ＜0.001 | 1.20(1.15-1.25) | ＜0.001 |
| Heart disease | | | | | | | |
| Sleep quality |  |  | ＜0.001 |  | ＜0.001 |  | ＜0.001 |
| good | 694/4662 | Reference |  | Reference |  | Reference |  |
| fair | 462/2691 | 1.19(1.04-1.35) | 0.01 | 1.15(1.01-1.30) | 0.04 | 1.19(1.04-1.36) | 0.01 |
| bad | 306/1239 | 1.88(1.61-2.18) | ＜0.001 | 1.78(1.52-2.07) | ＜0.001 | 1.80(1.53-2.11) | ＜0.001 |
| Continuous | 1462/8592 | 1.34(1.24-1.44) | ＜0.001 | 1.30(1.21-1.40) | ＜0.001 | 1.32(1.28-1.36) | ＜0.001 |
| Stroke | | | | | | | |
| Sleep quality |  |  | ＜0.001 |  | ＜0.001 |  | ＜0.001 |
| good | 450/4662 | Reference |  | Reference |  | Reference |  |
| fair | 286/2691 | 1.11(0.95-1.30) | 0.18 | 1.13(0.97-1.32) | 0.13 | 1.14(0.97-1.34) | 0.11 |
| bad | 171/1239 | 1.59(1.24-1.81) | ＜0.001 | 1.52(1.25-1.84) | ＜0.001 | 1.48(1.22-1.80) | ＜0.001 |
| Continuous | 907/8592 | 1.20(1.10-1.32) | ＜0.001 | 1.21(1.11-1.33) | ＜0.001 | 1.21(1.16-1.25) | ＜0.001 |
| Dyslipidemia | | | | | | | |
| Sleep quality |  |  | ＜0.001 |  | ＜0.001 |  | ＜0.001 |
| good | 249/4662 | Reference |  | Reference |  | Reference |  |
| fair | 144/2691 | 1.00(0.81-1.24) | 0.99 | 0.97(0.79-1.21) | 0.81 | 1.04(0.83-1.30) | 0.71 |
| bad | 105/1239 | 1.64(1.29-2.08) | ＜0.001 | 1.55(1.22-1.98) | ＜0.001 | 1.60(1.24-2.06) | ＜0.001 |
| Continuous | 498/8592 | 1.23(1.10-1.39) | ＜0.001 | 1.20(1.06-1.36) | ＜0.001 | 1.24(1.17-1.30) | ＜0.001 |

Notes: The table uses the dataset after multiple imputation. Model 1 no adjusted, Model 2 adjusted for age and sex, Model 3 further adjusted for place of residence, educational attainment, living arrangement, marital status, occupation, smoking, drinking, exercise, and body mass index.

**Table S3.** The association between frailty and cardiometabolic multimorbidity(N=8592)

| **Outcome** | **Events/No.** | **Model 1** |  | **Model 2** |  | **Model 3** |  |
| --- | --- | --- | --- | --- | --- | --- | --- |
|  |  | **OR(95%CI)** | **P-value** | **OR(95%CI)** | **P-value** | **OR(95%CI)** | **P-value** |
| Cardiometabolic multimorbidity | | | | | | | |
| Frailty |  |  | ＜0.001 |  | ＜0.001 |  | ＜0.001 |
| non-frail | 1329/7399 | Reference |  | Reference |  | Reference |  |
| frail | 525/1193 | 3.59(3.16-4.08) | ＜0.001 | 6.39(5.48-7.45) | ＜0.001 | 6.98(5.90-8.25) | ＜0.001 |
| Hypertension | | | | | | | |
| Frailty |  |  | ＜0.001 |  | ＜0.001 |  | ＜0.001 |
| non-frail | 2942/7399 | Reference |  | Reference |  | Reference |  |
| frail | 679/1193 | 2.00(1.77-2.27) | ＜0.001 | 2.73(2.39-3.13) | ＜0.001 | 2.68(2.32-2.08) | ＜0.001 |
| Diabetes | | | | | | | |
| Frailty |  |  | ＜0.001 |  | ＜0.001 |  | ＜0.001 |
| non-frail | 642/7399 | Reference |  | Reference |  | Reference |  |
| frail | 214/1193 | 2.30(1.94-2.72) | ＜0.001 | 3.97(3.29-4.79) | ＜0.001 | 3.73(3.05-4.56) | ＜0.001 |
| Heart disease | | | | | | | |
| Frailty |  |  | ＜0.001 |  | ＜0.001 |  | ＜0.001 |
| non-frail | 1042/7399 | Reference |  | Reference |  | Reference |  |
| frail | 420/1193 | 3.32(2.89-3.80) | ＜0.001 | 4.36(3.76-5.07) | ＜0.001 | 4.32(3.68-5.07) | ＜0.001 |
| Stroke | | | | | | | |
| Frailty |  |  | ＜0.001 |  | ＜0.001 |  | ＜0.001 |
| non-frail | 575/7399 | Reference |  | Reference |  | Reference |  |
| frail | 332/1193 | 4.58(3.93-5.33) | ＜0.001 | 6.75(5.68-8.01) | ＜0.001 | 6.73(5.61-8.08) | ＜0.001 |
| Dyslipidemia | | | | | | | |
| Frailty |  |  | ＜0.001 |  | ＜0.001 |  | ＜0.001 |
| non-frail | 400/7399 | Reference |  | Reference |  | Reference |  |
| frail | 98/1193 | 1.57(1.24-1.97) | ＜0.001 | 2.48(1.94-3.17) | ＜0.001 | 2.41(1.84-3.15) | ＜0.001 |

Notes: The table uses the dataset after multiple imputation. Model 1 no adjusted, Model 2 adjusted for age and sex, Model 3 further adjusted for place of residence, educational attainment, living arrangement, marital status, occupation, smoking, drinking, exercise, and body mass index.

**Table S4.** The characteristics of the research subjects after multiple fillings

| **Variable** | **Total(N=8592)** | **CMM group**  **(n=1854)** | **Non-CMM group**  **(n=6738)** | **P-value** |
| --- | --- | --- | --- | --- |
| Age, No. (%) | | | | ＜0.001 |
| 65-74 | 2206(25.6) | 541(29.2) | 1665(24.7) |  |
| 75-84 | 2651(30.9) | 688(37.1) | 1963(29.2) |  |
| 85-94 | 2161(25.2) | 455(24.5) | 1706(25.3) |  |
| ≥95 | 1574(18.3) | 170(9.2) | 1404(20.8) |  |
| Sex, No. (%) | | | | 0.01 |
| male | 4126(48.0) | 839(45.3) | 3287(48.8) |  |
| female | 4466(52.0) | 1015(54.7) | 3451(51.2) |  |
| Marital status, No. (%) | | | | ＜0.001 |
| married | 3935(45.8) | 955(51.5) | 2980(44.2) |  |
| partnered/single/widowed | 4657(54.2) | 899(48.5) | 3758(55.8) |  |
| Residence, No. (%) | | | | ＜0.001 |
| city | 1975(23.0) | 784(42.3) | 1191(17.7) |  |
| town | 2858(33.3) | 488(26.3) | 2370(35.2) |  |
| rural | 3759(43.7) | 582(31.4) | 3177(47.1) |  |
| Body Mass Index (BMI), No. (%) | | | | ＜0.001 |
| ＜18.5 | 1239(14.5) | 114(6.1) | 1125(16.7) |  |
| 18.5-24 | 4512(52.5) | 830(44.8) | 3682(54.6) |  |
| 24-28 | 2116(24.6) | 662(35.7) | 1454(21.6) |  |
| ＞28 | 725(8.4) | 248(13.4) | 477(7.1) |  |
| Co-residence, No. (%) | | | | ＜0.001 |
| living with house member(s) | 6944(80.8) | 1493(80.5) | 5451(80.9) |  |
| living alone | 1423(16.6) | 289(15.6) | 1134(16.8) |  |
| in an institution | 226(2.6) | 72(3.9) | 154(2.3) |  |
| Smoking, No. (%) | | | | ＜0.001 |
| yes | 1422(16.6) | 219(11.8) | 1203(17.9) |  |
| no | 7170(83.4) | 1635(88.2) | 5535(82.1) |  |
| Drinking, No. (%) | | | | ＜0.001 |
| yes | 1370(15.9) | 191(10.3) | 1179(17.5) |  |
| no | 7222(84.1) | 1663(89.7) | 5559(82.5) |  |
| Physical activity, No. (%) | | | | ＜0.001 |
| yes | 3044(35.4) | 801(43.2) | 2243(33.3) |  |
| no | 5548(64.6) | 1053(56.8) | 4495(66.7) |  |
| Occupation, No. (%) | | | | ＜0.001 |
| non-farmer | 3253(37.9) | 1038(56.0) | 2215(32.9) |  |
| farmer | 5339(62.1) | 816(44.0) | 4523(67.1) |  |
| Education, No. (%) | | | | ＜0.001 |
| primary | 6767(78.7) | 1239(66.8) | 5528(82.1) |  |
| secondary | 1500(17.5) | 459(24.8) | 1041(15.4) |  |
| tertiary | 325(3.8) | 156(8.4) | 169(2.5) |  |

Note: CMM = Cardiometabolic multimorbidity.

**Table S5.** The combined effect of sleep quality and frailty on cardiometabolic multimorbidity and its components after multiple imputation

|  | **CMM** | |  | **Heart disease** | |
| --- | --- | --- | --- | --- | --- |
| good | Reference | 7.57(5.96-9.62) |  | Reference | 4.43(3.51-5.60) |
| fair | 1.13(0.98-1.31) | 7.10(5.52-9.12) |  | 1.14(0.97-1.33) | 4.41(3.45-5.64) |
| bad | 1.65(1.37-1.98) | 9.31(6.95-12.46) |  | 1.56(1.29-1.90) | 6.07(4.60-8.01) |
|  | **Hypertension** | |  | **Stroke** | |
| good | Reference | 2.19(1.80-2.67) |  | Reference | 5.87(4.62-7.44) |
| fair | 1.09(0.97-1.21) | 2.68(2.17-3.31) |  | 1.09(0.89-1.32) | 5.52(4.27-7.44) |
| bad | 1.44(1.24-1.66) | 2.85(2.20-3.71) |  | 1.25(0.98-1.61) | 6.31(4.70-8.46) |
|  | **Diabetes** | |  | **Dyslipidemia** | |
| good | Reference | 2.74(2.08-3.61) |  | Reference | 1.93(1.25-2.97) |
| fair | 1.19(0.99-1.44) | 3.20(2.40-4.26) |  | 0.94(0.73-1.20) | 2.60(1.72-3.93) |
| bad | 1.38(1.09-1.75) | 3.41(2.45-4.73) |  | 1.41(1.06-1.89) | 3.19(2.07-4.93) |
|  | non-frail | frail |  | non-frail | frail |

Note: CMM = Cardiometabolic multimorbidity.

**Table S6.** Stratified analysis of the association between frailty and cardiometabolic multimorbidity (CMM) by sex and age group

| **subgroup** | **No. of participants** | **No. of CMM cases** | **aOR (95% CI)** | **P–value** | **P for interaction** |
| --- | --- | --- | --- | --- | --- |
| Sex |  |  |  |  | 0.05 |
| Male | 4126 | 839 | 7.41 (5.64-9.75) | <0.001 |  |
| Female | 4466 | 1015 | 6.41 (4.97-8.28) | <0.001 |  |
| Age group |  |  |  |  | <0.001 |
| <85 years | 4857 | 1229 | 9.87 (7.27-13.39) | <0.001 |  |
| ≥85 years | 3735 | 625 | 4.52 (3.59-5.68) | <0.001 |  |

Note: Adjusted for all covariates in Model 3 except the stratification variable itself. For sex‑stratified analysis, adjustment included age, place of residence, educational attainment, living arrangement, marital status, occupation, smoking, drinking, exercise, and body mass index. For age‑stratified analysis, adjustment included sex, and the same set of other covariates.
